# The Development of Serum Therapy

**Published:** 1898-10

**Authors:** Chas. T. M’Clintock

**Affiliations:** Detroit, Mich.


For the Texas Medical Journal.

### The Development of Serum Therapy.

BY CHAS. T. M'CLINTOCK, DETROIT, MICH.

[REPRINT.]

Natural immuuity, the fact that certain individuals or races are less susceptible than others to certain diseases, was noted by the earliest writers on medicine.

So it has long been known that in some of the infectious diseases one attack of the disease confers more or less immunity against subsequent attacks—an acquired immunity.

Artifically acquired immunity dates from the discovery of Jenner (1766) that the lymph taken from the vesicles of cow pox would protect the vaccinated individual from small pox.

Pasteur[1] in 1880 began the publication of a series of brilliant experiments on the production of artificial immunity. He found that if his microbes were weakened by age, or heat, or exposure to the atmosphere, animals inoculated by these weakened cultures would have a mild attack of the disease and were thereafter immune to the disease in question even after inoculation with the most virulent cultures.

He perfected his method of attenuating his microbes and succeeded in immunizing animals against several of the infectious cases.

Pasteur clearly saw the immense significance of the work and made the prediction: "It is possible for man to eradicate every contagious disease from off the face of the earth."

The epoch-making discoveries of Pasteur attracted many workers to this field and justified the statement of one of his pupils, "There are two periods in the history of medicine, the one before, the other after, Pasteur."

Pasteur worked with the germs themselves. The next discovery of importance was that the toxic products of the germs, the filtered cultures from which all the germs were removed, could produce immunity. This discovery was made piecemeal. The first satisfactory demonstration of it was given by Salmon and Smith[2] in 1886. These workers succeeded in immunizing pigeons against the germs of hog cholera by previous injections of the sterilized cultures obtained from these germs.

Similar results were reported by Roux[3] in 1888 with anthrax and symptomatic anthrax. The first published results on immunization in diphtheria were those of Loeffler.[4] He showed that in May, 1888, he had inoculated a guinea pig with diphtheria. The animal sickened, but recovered after three weeks. It was then inoculated a number of times with virulent cultures, but showed itself entirely immune.

C. Fraenkel (*Berlin Klin. Woch.*, December 3, 1890) published his results on the immunization of animals against diphtheria. He showed that there were several ways in which this could be accomplished.

On the following day (*Deutsch. Med. Woch.*, December 4, 1890) Behring and Kitasato published their results on the immunization against diphtheria and tetanus.

They succeeded in immunizing animals against tetanus and diphtheria by the injection of the filtered cultures from these germs. In 1891 the Klemperer Brothers[5] reported similar results with pneumonia. Meantime a number of investigators in various parts of the world were busy with another part of the problem.

As early as 1872 Lewis and Cunningham[6] demonstrated the fact that bacteria injected into the circulation rapidly disappear.

In 1874, Traube and Gschiedlen[7] found that arterial blood taken under antiseptic precautions from a rabbit into the jugular vein of which 1½ Cc. of a fluid rich in putrefactive germs had been injected forty-eight hours previously, failed to undergo decomposition for months. These investigators attributed the germicidal properties of the blood to its ozonized oxygen. Similar results were obtained by Fodor[8] and by Wysokowicz.[9] The latter accounted for the disappearance of the germs, not by supposing that they were destroyed by the blood, but that they found lodgment in the capillaries.

The first experiments made with the extra-vascular blood were conducted by Grohmann[10] under the direction of A. Schmidt in his researches upon the cause of coagulation. It was found that anthrax bacilli, after being kept in plasma, were less virulent, as was demonstrated by their effect upon rabbits. Grohmann supposed that in some way the bacteria were influenced by the process of coagulation.

In 1877 Fodor[11] made a second contribution to this subject and in this he combated the retention theory of Wysokowicz. One minute after the injection of 1 Cc. of anthrax culture into the jugular vein, in eight samples of blood, Fodor found only one colony of the bacillus. Then he took the blood from the heart with a sterilized pipette and added anthrax bacilli to it. This was kept at 39° C., and plates made from time to time showed a rapid diminution in the number of germs; after a time, when the blood had lost its germicidal properties, the number of germs began to increase.

In 1888, Nuttall[12] showed that defibrinated blood had this germicidal power. Since then Nissen,[13] Behring,[14] Buchner,[15] Christmas,[16] Hankin,[17] Bitter,[18] and others have worked on this question. All of these observers find that the blood, within or without the body, has germicidal powers.

These experiments demonstrated the fact that the body had the power to kill at least some bacteria, but gave us little if any insight into how it was done.

IMMUNITY TO POISONS.

That certain animals possesed more or less immunity to certain poisons was a matter of common knowledge. The carniverous animals as the wolf and vulture thriving on putrid, toxic flesh. So it was known that many snakes and a few mammals, as the hedgehog, swine and the ichneumon, were very resistant to snake poison.

In 1887, Sewall[19] reported that he had immunized pigeons against the poison of the rattlesnake. He says: "This work was undertaken with the hope that it might form a worthy contribution to the theory of prophylaxis. I have assumed an analogy between the venom of poisonous serpents and the ptomaines produced under the influence of bacterial organism." These experiments of Sewall's, are in so far as we know, the first attempt to immunize animals against poison.

An all important discovery was that the immunity of one animal could be borrowed and transferred to another animal. The idea of the transfusion of the blood of healthy individuals or animals into persons weakened by disease or age is centuries old. King, physician to King Charles, in 1665, practiced transfusion from the veins of one person into that of another. Denis in France, who died in 1704, transfused the blood of a lamb into a weak patient. This operation was in common use until within the last few years. The first record we find of the use of the blood of an immunized animal for the protection of other animals is in 1887. Foa and Bonome[20] published their results. They rendered animals immune to the proteus vulgaris, the diplococcus of pneumonia, and the bacillus of chicken cholera, by treating them with sterilized cultures of germs, and discovered that the blood drawn from the heart, or an infusion of the tissues of rabbits dead from proteous infection, injected intravenously into another rabbit, made this animal immune to virulent cultures of the proteous germs.

Hericourt and Richet,[21] 1888, report treating dogs with tubercle germs, and then transferring the blood from these dogs into other animal.to cure and prevent the disease.

So they injected cultures of staphylococcus pyosepticus into dogs, taking the blood from these dogs and injecting it into rabbits they found that the latter animals would resist infection with the stapylococcus.

In 1890, Ogata and Jasuhara,[22] working in the Hygienic Institute of Tokio, showed that mice inoculated with a lethal dose of anthrax germs could be saved if at the same time blood from an immune animal was injected subcutaneously.

Later in the year (1890), Behring and Kitasato[23] showed "that the blood of an animal which has acquired immunity against tetanus or diphtheria, when added to a virulent culture of one or the other of these bacilli, neutralizes the pathogenic power of such cultures, as shown by inoculation into susceptible animals." And also that cultures from which the bacilli have been removed by filtration, and which kill susceptible animals in very small amounts, have their toxic potency destroyed by adding to them the blood of an immune animal.

In the experiment cited of Fraenkel, Behring and Kitasato, it was clearly shown that the blood of immunized animals when injected into susceptible animals protected them from subsequent infection with virulent germs. Behring and Kitasato went further and demonstrated that the immunized blood would protect animals not only against living germs but also against filtered cultures or toxins, which were very fatal to untreated animals. They showed that their protective inoculations produced an immunity which in mice lasted for forty or fifty days, after which it was gradually lost.

The next step forward was made by Kitasato[24] in 1891, in his further research on tetanus. He discovered that the blood serum of animals immunized to tetanus had not only prophylactic but also curative properties. He demonstrated that mice inoculated with tetanus germs could be cured by an injection of serum, even *after* the tetanus symptoms had appeared.

This, the last in the long list of discoveries made by many workers along this line, made serum therapy possible. From this time on the question was one of perfection of methods. The best animals to use, methods for so increasing the immunizing power of the blood that a small quantity would suffice for curative purposes, methods for determining the strength of the immunizing serum, how to preserve and administer it.

Many investigators assisted in this work. Ehrlich[25] in his work on the vegetable poisons, abrin and ricin, showed that by beginning with very small doses and gradually increasing them, the immunizing power of the blood can be increased almost indefinitely. His treated animals would stand hundreds of times the fatal dose of the poison. Behring contributed largely to the work, and devised a method for measuring the immunizing strength of the antitoxic serum.

Roux and Yersin[26] showed that different cultures of diphtheria germs varied enormously in virulence.

Aronson[27] and others devised means for increasing the virulence and toxicity of the germs. Aronson[28] after experimenting with many species of animals, could show that the horse was the most suitable for the production of antitoxic serum.

The possibilities of the serum therapy was apparent to every worker. Roux[29] in 1894, when announcing the successful results of the treatment of children with his antitoxin, says, "Since the year 1891 we have carried on experiments on tne treatment of diphtheria with antitoxic serum."

Behring and Wernicke[30] (in 1892) published a paper upon the immunizing and healing of experimental animals with diphtheria. They produced an antitoxin and used this in treating guinea pigs, both for immunizing these animals and for curing them after they have been infected with the diphtheria germ, or with its poison. They say the aim of their experiments "is to produce the material in such quantity and potency that diphtheria in human beings can be treated therewith." Behring, in his History of Diphtheria, 1893, discussed the possibility of treating children with diphtheria antitoxin, pointing out that it is only a question of making the material more potent until the work can be begun. He shows that of the material produced up to that time about 50 Cc. would have to be given to a child of 20 kilogramme weight, supposing that diphtheria in children is of about the same intensity as in guinea pigs.

In November, 1892, in the Berlin Medical Society, Aronson[31] reported on the immunization of human beings against diphtheria, and in January, 1893,[32] he further reported that he had succeeded for the first time in so increasing the immunizing power of the serum that protective inoculation of children exposed to diphtheria was possible in practice.

In March, 1894, Aronson's serum began to be used in the Children's Hospital in Berlin. In February, 1894, Roux's serum began to be used in one of the hospitals of Paris, his preliminary report on the clinical results being made in May, 1894.

In September, 1894, in Buda Pesth, at the International Congress of Hygiene, papers were read by Behring and by Roux on the treatment of human diphtheria with antitoxin. These papers with their clinical reports of the value of the treatment at once aroused world-wide attention.

As so often happens, the persons who presented the results were popularly supposed to be the sole discoverers. On the contrary, antitoxin was not the discovery of any one man or set of men, but the cumulative result of the work of many investigators. The papers cited herein are but a few of the hundreds that bear on this question.

BIBLIOGRAPHY.

[1]Pasteur: De l'attenuation du virus du cholera des poules. Compte rendu, Acad. des Sci., t. xci., 1880, p. 673.

[2]Salmon and Smith: Experiments on the Production of Immunity by Hypodermic Injection of Sterilized Cultures. Centralbl. fur Bakteriol., bd. II., 1887, p. 543.

[3]Roux: Immunite contre le carbon symptomatique confere par les substances solubles. Annales de l'Institut Pasteur, 1888.

[4]Loffler: Deutsche Med Woch., 1890, Nos. 5 and 6.

[5]Klemperer, G. and F.: Ueber die Heilung von Infectionskrankheiten durch nachtragliche Immunisirung. Berl. Klin. Woschr., 1892, p. 421.

[6]Lewis and Cunningham: Eighth Annual Report of the Sanitary Commission of the Government of India.

[7]Traube and Gschiedlen: Schlesische Gesellschaft f. Vaterland. Cultur, 1874.

[8]Fodor: Archiv. f. Hygiene, B. 4.

[9]Wysokowicz; Zeitschrift f. Hygiene, B. I.

[10]Grohmann: Ueber die Einwirkung des zellenfreien Blutplasma auf einige pflanziche Mikroorganismen, Dorpat, 1884.

[11]Fodor: Deutsche Medicinische Wochenschrift, 1887.

[12]Nuttall: Zeitschrift f. Hygiene, B. 4.

[13]Nissen: Ibid., B. 6.

[14]Behring: Ibid., B. 6.

[15]Buchner: Archiv. f. Hygiene, B. 10.

[16]Christmas: Annales de l'Institut Pasteur, t. v.

[17]Hankin: Centralblatt f. Bakteriologie, B. 9.
[18]Bitter; Zeitschrift f. Hygiene, B. 12.
[19]Sewell: Jour. Physiology, vol. viii , 1887, p. 203.
[20]Foa and Bonome: Zeits f. Hygiene, 5, 415.
[21]Hericourt and Richet: Abstract im Deutsch Med. Ztg., 1888, p. 1141.
[22]Ogata and Jasuhara: Ueber die Einflusse einiger Thierblutarten auf Milzbrandbacillen. Centralbl. fur Bacteriol., Bd., ix, p. 25.
[23]Behring and Kitasato: Ueber das Zustandekommen der Diptherie Immunitat und der Tetanus-immunitat bei Thieren. Deutsche Med. Wchschr., 1890, No. 49.
[24]Kitasato: Experimentelle Untersuchungen uber das Tetanusgift Zeitschr. f. Hygiene, 1891.
[25]Ehrlich: Experimentelle Untersuchungen uber Immunitat. Deutsch. Med. Wchschr., 1891, No. 32; Ibid., No. 44.
[26]Roux and Yersin: Annales de l'Institut Pasteur, 1890.
[27]Aronson: Berlin Klin. Woch., 1894, Nos. 18 and 19.
[28]Aronson: Berlin Klin. Woch., 1894, Nov.
[29]Roux: Annales de l'Institut Pasteur, 1894.
[30]Behring and Wernicke: Zeitschr. f. Hygiene, 1892.
[31]Aronson: Berlin Klin. Woch., 1893, p. 100.
[32]Aronson: Berlin Klin. Woch., 1893, p. 215.

